# Ultrastructural Characterization of Pandemic (H1N1) 2009 Virus

**DOI:** 10.3201/eid1711.110258

**Published:** 2011-11

**Authors:** Cynthia S. Goldsmith, Maureen G. Metcalfe, Dominique C. Rollin, Wun-Ju Shieh, Christopher D. Paddock, Xiyan Xu, Sherif R. Zaki

**Affiliations:** Centers for Disease Control and Prevention, Atlanta, Georgia, USA

**Keywords:** viruses, pandemic, pandemic (H1N1) 2009, influenza, electron microscopy, immunoelectron microscopy, viral proteins, dispatch

## Abstract

We evaluated pandemic influenza A (H1N1) 2009 virus isolates and respiratory tissues collected at autopsy by electron microscopy. Many morphologic characteristics were similar to those previously described for influenza virus. One of the distinctive features was dense tubular structures in the nuclei of infected cells.

In April 2009, a novel influenza A (H1N1) virus was first detected in 2 children in California; the same virus was found to be circulating in Mexico and then spread rapidly worldwide (*1*). The virus became known as pandemic (H1N1) 2009 and generally caused a mild-to-moderate illness, although severe and fatal cases were reported.

Influenza A virus is a member of the family *Orthomyxoviridae* and contains a genome that is composed of single-stranded negative-sense RNA that, with the viral nucleoprotein, is formed into 8 separate ribonucleoprotein segments (*2*). The pandemic (H1N1) 2009 virus contains a unique combination of RNA segments from North American and Eurasian swine lineages and is capable of human-to-human transmission (*3*).

The pathologic features of fatal cases of pandemic (H1N1) 2009 have been described (*4**,**5*). Notably, in addition to infection of the tracheobronchial epithelium, as is seen with seasonal influenza, pandemic (H1N1) 2009 virus also extensively infects the lower respiratory system. The most common histopathologic finding was diffuse alveolar damage comprising intraalveolar edema, hyaline membranes, fibrin, and hemorrhage. Immunohistochemical examination detected influenza virus antigens in type II pneumocytes, in epithelial cells in the upper airways, and in submucosal glands.

In this study, we examined the morphologic properties of pandemic (H1N1) 2009 virus in cultured cells and in human tissues obtained at autopsy. Immunogold labeling was used to further analyze various aspects of the morphogenesis of this novel influenza virus.

## The Study

Infected and uninfected MDCK cells were embedded for standard electron microscopy or immunogold electron microscopy in a mixture of Epon-substitute and Araldite or in LR White resin (Ted Pella, Inc., Redding, CA, USA), respectively, as described (*6*). The immunogold electron microscopy protocol used a goat antibody raised against the matrix protein of influenza A virus as a primary antibody and a donkey anti-goat antibody conjugated to 12-nm colloidal gold particles as a secondary antibody. In addition, lung tissues were obtained from the upper and lower respiratory tracts of 2 patients who died of pandemic (H1N1) 2009. Samples from each patient were negative for parainfluenza viruses and respiratory syncytial virus by PCR. Areas for examination were selected on the basis of strong immunohistochemical labeling for influenza virus. Sections for light microscopy sections were cut from the electron microscopy blocks, and areas were selected for either bronchus with submucosal glands or lung with alveoli.

**Figure 1:**
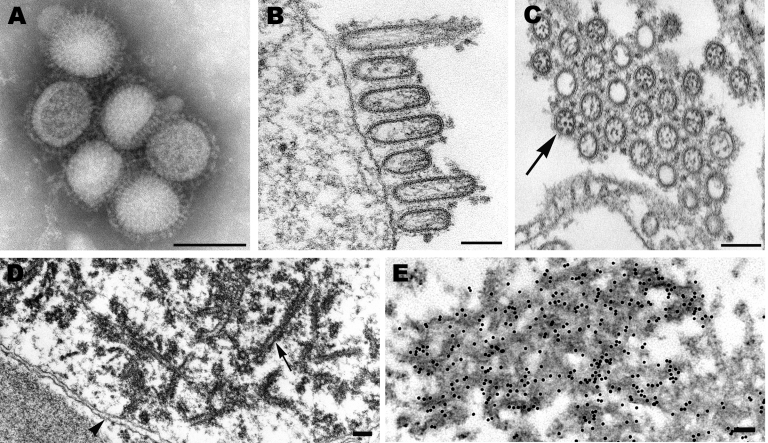
Electron microscopy of pandemic (H1N1) 2009 virus. A) Negatively stained virions grown in MDCK cells showing spherical particles with distinct surface projections. Scale bar = 100 nm. B) Filamentous and ovoid particles assembling at the plasma membrane. Scale bar = 100 nm. C) Extracellular particles showing internal nucleocapsids, seen in cross-section, surrounded by an envelope with prominent spikes. Note all 8 nucleocapsids present in 1 virion (arrow). Scale bar = 100 nm. D) Dense tubules (arrow), which were found in the nuclei of some MDCK-infected cells. Arrowhead, nuclear envelope. Scale bar = 100 nm. E) Immunogold labeling of the nuclear tubules by using an antibody against the matrix protein. Scale bar = 100 nm.

Pandemic (H1N1) 2009 virus isolates grown in MDCK cells were morphologically similar to those of other influenza A viruses. In negative stain preparations, virions appeared mostly spherical (average diameter 104 nm) with some filamentous particles (up to 3.3 μm in length) and contained surface projections of the hemagglutinin and neuraminidase glycoproteins (Figure 1, panel A). By thin section electron microscopy, infected cells showed virus particles being assembled mostly at the plasma membrane. Extracellular virions, averaging 86 nm in diameter, were mostly ovoid or filamentous (Figure 1, panel B). The virions were surrounded by the glycoprotein spikes, and the individual nucleocapsids inside the virions were seen as thin threads (when cut longitudinally) or dark dot-like figures (when cut in cross-section) and measured ≈8 nm in diameter (Figure 1, panels B and C).

The nuclei of some infected cells contained dense tubular structures, which had a rough outer edge and averaged 37 nm in width (Figure 1, panel D). Immunogold electron microscopy labeling that used an anti–matrix protein goat antibody detected matrix (M) proteins on the dense tubular structures (Figure 1, panel E) as well as on virions (C. Goldsmith et al., unpub. data).

**Figure 2:**
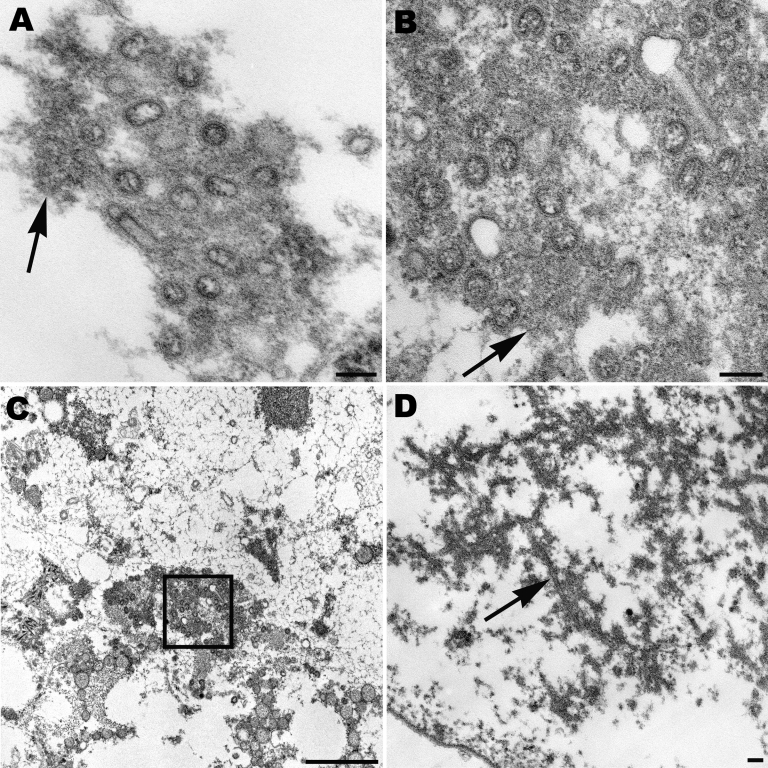
Spherical and ovoid extracellular pandemic (H1N1) 2009 virus particles in human lung tissue found in the alveolar space (A) and in a submucosal gland (B). Nucleocapsids and surface projections are visible on some virions. Note the dense material (arrows) associated with the particles. Scale bars = 100 nm. C) Low-power magnification of the aggregation of virus particles seen in panel B, showing virions (box) in the mucus of the submucosal gland. Scale bar = 1 μm. D) Dense tubules (arrow) found in the nucleus of an infected cell in alveolar space. Scale bar = 100 nm.

Lung tissues from patients with fatal cases of pandemic (H1N1) 2009 were examined by electron microscopy. Infected cells and virions were observed in the alveolar spaces and in the submucosal glands (Figure 2, panels A, B, and C). Spherical or ovoid extracellular viral particles, which may represent cross-sections through filamentous particles, were seen in respiratory tissues. Dense, amorphous material was associated with the virions. In addition, intranuclear dense tubules, similar to those seen in tissue culture–infected cells, were recognized in infected cells (Figure 2, panel D).

## Conclusions

This study shows that the morphologic features of pandemic (H1N1) 2009 virus in infected cells were similar to other members of the family *Orthomyxoviridae* (*7*). Enveloped particles, either spherical or filamentous, were surrounded by a fringe of surface projections and enclosed viral nucleocapsids. As is typical for influenza viruses, virions assembled and budded at the plasma membrane. In addition, inclusions consisting of dense tubules were seen in the nuclei of some infected cells in cell culture and tissues collected during autopsy. Although intranuclear dense tubules have been previously reported for other influenza A-infected cells, these were either smooth or helical (*8**,**9*). Of note, the dense tubules in the nuclei of pandemic (H1N1) 2009–infected cells were much larger (37 nm vs. 8 nm) and did not resemble the more thread-like nucleocapsids of influenza virus.

Of particular interest is the labeling of the nuclear dense structures in pandemic (H1N1) 2009–infected cells by an antibody directed against the matrix protein. M1 serves as the matrix protein during the formation of viral particles. The M1 protein is also transported from the cytoplasm into the nucleus during viral replication and, in conjunction with nonstructural protein 2, is involved in the export of the ribonucleoproteins out of the nucleus (*10**,**11*). The immunolabeling of the dense tubular structures seen in the pandemic (H1N1) 2009–infected cells reported here confirms the presence of matrix protein in the nuclei of infected cells.

Generally, freshly isolated viruses from human tissues are predominantly filamentous, whereas laboratory-adapted strains are predominantly spherical (*12*). This study and other recent reports of early isolates of pandemic (H1N1) 2009 revealed both spherical and filamentous forms (*4**,**13*). Similarly, in this study, mostly spherical particles were found in lung autopsy tissues, whereas filamentous particles were as described by Nakajima et al. (*4*). The determinants of influenza virus morphology, i.e., spherical versus filamentous, have been evaluated by many researchers and found to be influenced by the M1 and M2 proteins and by polarization of the host cell (*14**,**15*). The biological relevance of finding both spherical and filamentous particles in autopsy tissues and in early isolates of pandemic (H1N1) 2009 virus requires further studies.

Another distinctive feature of pandemic (H1N1) 2009 virus was infection of the lower respiratory tract, as evidenced by the presence of viral particles in the alveoli. This finding helps explain the diffuse alveolar damage associated with hyaline membranes seen in severe cases of infection. Pandemic (H1N1) 2009 virus particles were also found in the mucus of the submucosal glands and may play a major role in human-to-human transmission through aerosolization of respiratory secretions.

Pandemic (H1N1) 2009 virus was the causative agent of the first influenza pandemic since 1968, and much has been learned since the pandemic began. However, there is still much to be elucidated about this emerging virus, and electron microscopic studies have revealed distinctive features of the pandemic (H1N1) 2009 virus that may help in understanding its morphogenesis and pathogenesis.
